# Advanced Meta-Heuristics, Convolutional Neural Networks, and Feature Selectors for Efficient COVID-19 X-Ray Chest Image Classification

**DOI:** 10.1109/ACCESS.2021.3061058

**Published:** 2021-02-22

**Authors:** El-Sayed M. El-Kenawy, Seyedali Mirjalili, Abdelhameed Ibrahim, Mohammed Alrahmawy, M. El-Said, Rokaia M. Zaki, Marwa Metwally Eid

**Affiliations:** 1 Department of Communications and ElectronicsDelta Higher Institute of Engineering and Technology (DHIET) Mansoura 35111 Egypt; 2 Centre for Artificial Intelligence Research and OptimizationTorrens University Australia 386703 Fortitude Valley QLD 4006 Australia; 3 Yonsei Frontier LabYonsei University 26721 Seoul 03722 South Korea; 4 Computer Engineering and Control Systems DepartmentFaculty of EngineeringMansoura University 68779 Mansoura 35516 Egypt; 5 Department of Computer ScienceFaculty of Computers and InformationMansoura University 68779 Mansoura 35516 Egypt; 6 Electrical Engineering DepartmentFaculty of EngineeringMansoura University 68779 Mansoura 35516 Egypt; 7 Delta Higher Institute of Engineering and Technology (DHIET) Mansoura 35111 Egypt; 8 Department of Electrical EngineeringShoubra Faculty of EngineeringBenha University 68816 Benha 11629 Egypt

**Keywords:** Chest X-ray, transfer learning, convolutional neural network, squirrel search optimization, multilayer perceptron, optimization algorithm

## Abstract

The chest X-ray is considered a significant clinical utility for basic examination and diagnosis. The human lung area can be affected by various infections, such as bacteria and viruses, leading to pneumonia. Efficient and reliable classification method facilities the diagnosis of such infections. Deep transfer learning has been introduced for pneumonia detection from chest X-rays in different models. However, there is still a need for further improvements in the feature extraction and advanced classification stages. This paper proposes a classification method with two stages to classify different cases from the chest X-ray images based on a proposed Advanced Squirrel Search Optimization Algorithm (ASSOA). The first stage is the feature learning and extraction processes based on a Convolutional Neural Network (CNN) model named ResNet-50 with image augmentation and dropout processes. The ASSOA algorithm is then applied to the extracted features for the feature selection process. Finally, the Multi-layer Perceptron (MLP) Neural Network’s connection weights are optimized by the proposed ASSOA algorithm (using the selected features) to classify input cases. A Kaggle chest X-ray images (Pneumonia) dataset consists of 5,863 X-rays is employed in the experiments. The proposed ASSOA algorithm is compared with the basic Squirrel Search (SS) optimization algorithm, Grey Wolf Optimizer (GWO), and Genetic Algorithm (GA) for feature selection to validate its efficiency. The proposed (ASSOA + MLP) is also compared with other classifiers, based on (SS + MLP), (GWO + MLP), and (GA + MLP), in performance metrics. The proposed (ASSOA + MLP) algorithm achieved a classification mean accuracy of (99.26%). The ASSOA + MLP algorithm also achieved a classification mean accuracy of (99.7%) for a chest X-ray COVID-19 dataset tested from GitHub. The results and statistical tests demonstrate the high effectiveness of the proposed method in determining the infected cases.

## I. Introduction

Medical images are teeming with many features that can be considered for inspection. Generally, many processes in Computer-Aided System (CAD), such as pre-processing, isolating Regions of Interest (ROIs), and feature extracting process, can help to get the accurate classification of the diseases [1]. There are various approaches for highlighting ROIs, extracting the salient features, and suppressing the associated noises [2]–[4]. Rule-based techniques have limited performance, and to improve efficiency, they are usually consolidated. Traditional approaches focused on fetching geometric or handcrafted features are generally treated to reduce dimensionality, elapsed time, and redundancy features concerning extract salient features. Moreover, these methods suffer from failures that affect the classification accuracy. Hence, any improvements in the feature extraction step and the segmentation process are required [5]–[8]. For image classification tasks, some traditional classification methods have also achieved excellent results in recent years [9]–[12]; however, the deep learning methods have some advantages over the traditional methods. CNN’s (Convolutional Neural Networks) or pre-trained networks are commonly involved in different medical imaging tasks. They can offer rather good performance in analyzing high-resolution images as in abdominal X-rays. However, a need for sufficient amounts of the training dataset is a critical problem [13]–[15].

More training phases with large datasets are mainly required to apply the pre-trained networks for medical image classification tasks. Hence, in some cases, adopting these types of networks as classifiers are often not the preferred method to apply the CNN to the diagnosis tasks of CAD [16]–[18]. Using pre-trained networks (e.g., DCNNs), extra complicated datasets such as pneumonia’s presence/absence did not seem good. Therefore, more data augmentation samples and training can improve the efficiency [19], [20]. While identification systems based on CNN provide greater precision for various tasks, the key downside of these methods is the need for heavy training [21]–[23]. Hence, machine learning increases rapidly, which has caused many technical breakthroughs and is extensively employed in many fields. As a significant part of machine learning tasks, optimization has attracted much attention in several research areas. With the expedited growth of the amount of employed data and the increase of design complexity, optimization approaches in machine learning face more further challenges.

For a specific problem, optimization can be the most reliable solution between all available solutions, especially towards multi-dimensional space [24]. Practically, this involves maximization or minimization of an objective function. The objective function defines the solution candidate’s quality and efficiency represented by a particular vector in a search domain. There are two classes of optimization: nonlinear versus linear [25], [26]. Meta-heuristic algorithms are considered among the most powerful methods for solving real-world engineering problems [27]. Most of these algorithms’ derivation is done from physical algorithms’ rational behavior in nature, biological inspired algorithms’ behavior, swarm particles’ collective intelligence, and evolutionary algorithms’ fittest theory of survival [28], [29]. These optimization techniques provide acceptable solutions in a reasonable time with less computational effort. They are mostly used in engineering and science for finding solutions to complex and challenging problems because: a) of their utilization in different issues that come under other subjects, b) of no requirement of gradient information, c) they can bypass local optima, and d) they are easy to be implemented and are dependent on comparatively simple concepts.

This paper proposes a classification method to classify infected cases from the chest X-ray images. The method can decrease detection costs significantly. First, a feature learning stage is developed using the CNN model named ResNet-50 with image augmentation as a pre-processing and dropout as post-processing. Second, the features are extracted to start the feature selection process. A proposed Advanced Squirrel Search Optimization Algorithm (ASSOA) is developed for feature selection. The advanced classification stage starts to classify the infected cases using the optimized Multilayer Perceptron Neural Network (MLP) by the proposed ASSOA algorithm. ASSOA’s basic rule in the classification stage is to optimize the connection weights of MLP to improve accuracy. A dataset from Kaggle, chest X-ray images (Pneumonia) dataset [30] consists of 5,863 X-ray images used in experiments. A chest X-ray COVID-19 dataset [31] is also tested in the experiments. The proposed ASSOA algorithm is compared with the basic Squirrel Search (SS) optimization algorithm [32], Grey Wolf Optimizer (GWO) [29], and Genetic Algorithm (GA) [33] for feature selection to test its efficiency. The ASSOA + MLP algorithm is also compared with other classifiers, based on (SS + MLP), (GWO + MLP), and (GA + MLP), in performance metrics. Moreover, Wilcoxon rank-sum and one-way analysis of variance (ANOVA) are tested to statistically verify the proposed algorithm’s superiority.

This paper’s main contributions are as follows:

• An Advanced Squirrel Search Optimization Algorithm (ASSOA) is developed for feature extraction and classification.
• The proposed ASSOA algorithm adds horizontal, vertical, diagonal, and exponential movements to the basic moves in the search process of the basic SS algorithm.
• A new agents’ relocation equation is modeled in the proposed ASSOA algorithm, affecting local and global optima under specific conditions.
• A classification method for chest X-ray images is proposed based on the ASSOA algorithm.
• The classification method is tested using a dataset from Kaggle with 5,863 chest X-ray images.
• The classification method is also tested for a chest X-ray COVID-19 dataset from GitHub.
• Wilcoxon rank-sum and ANOVA statistical tests are performed to ensure the proposed ASSOA algorithm quality.

The next sections of this paper are as follows: Section II presents the related works. The materials and methods used in the study are defined in Section III. In-depth, Section IV describes the proposed method and the ASSOA algorithm. The experimental results are shown in Section V. Section IV discusses the proposed method findings. The research conclusions are seen in section VII.

## II. Related Work

Large datasets availability and the recent advances in deep learning models have led to the possession of power-assisted algorithms, which beats the medical professionals in various clinical image resolution. These images are such as cancer classification [34], detection of arrhythmia [35], [36], identification of haemorrhage [37], and diagnosis/detection of diabetic retinopathy [38]. Using radiography, the automated diagnosis of chest diseases has gained a lot of enthusiasm and interest. Several CNN models’ efficiency on various oddities certainly does not do well with all abnormalities, deep-learning approaches, and ensemble models may improve classification accuracy considerably reviewed to other Versions. Statistical dependence was studied between the precision levels of the predictions and the Multi-label Disease Classification (MDC). In the literature, the detection of health conditions from chest X-ray images was performed based on different methods [39]–[41]. The processes for X-ray cardiovascular angiogram images are proposed in the literature [42], [43].

Recent research has implemented several automatic pneumonia detection systems based on chest X-rays [44], [45]. Deep learning is applied for the training AI algorithms to detect pneumonia by studying chest X-ray images [46]. In terms of accuracy, Chhikara *et al.* achieved an accuracy of (90.1%) in [47] using 5,866 chest X-ray images compared to the latest models of classification. The CNN model proposed by Okeke Stephen *et al.* in [34], was constructed by extracting characteristics from the images of chest X-ray to test the existence of pneumonia. The authors in that model deployed multiple data augmentation algorithms to enhance both validation and classification accuracy of their model to achieve an accuracy of (93.73%).

An AI approach to diagnosing COVID-19 and other types of pneumonia is already developed in [48]. For COVID-19, their proposed method achieved an AUC (area under the curve) of (0.981) and accuracy of (92.49%). Butt *et al.* in [49] A CNN model called ResNet-18 was proposed to classify the CT images as COVID-19, regular, and pneumonia. With an AUC value of (0.996), they can achieve an accuracy of (86.7%). Authors in [50] The nCOVnet, based on deep learning, was proposed to detect COVID-19 by analyzing patients’ X-ray images. Their nCOVnet system obtained an AUC of (0.881) and a COVID-19 accuracy of (88.10%).

Nour *et al.* [51] using X-ray images, a CNN model trained from scratch was suggested. The model’s extracted features fed K-NN, SVM, and decision tree in their model. The SVM classifier achieved an accuracy of (98.97%). Hu *et al.* in [52] A weakly-supervised CNN model was proposed, which achieved an accuracy of (96.2%) with an AUC value of (0.970). To classify the chest’s x-ray images into COVID-19 or non-COVID-19, an ML-method in [53] was proposed. A Manta-Ray Foraging Optimization technique, using differential evolution, was developed for feature selection. The authors evaluated their method by testing two COVID-19 x-ray datasets. The recent machine learning research for CT and X-ray images is summarized in Table 1.

**TABLE 1:** Recent Machine Learning Research for Classification of CT and X-Ray Images

Reference	Methods	# of samples	# of classes	Type of Images	Accuracy	AUC
X. Wang et al. (2020) [52]	Weakly supervised deep learning model	450	3	CT	96.2%	0.970
K. Zhang et al. (2020) [48]	AI system based on ResNet-18	3,777	3	CT	92.49%	0.981
C. Butt et al. (2020) [49]	Multiple CNN models based on ResNet-18	618	3	CT	86.7%	0.996
O. Stephen et al. (2019) [34]	Efficient Deep Learning Approach	200	2	X-ray	93.73%	—-
D. Varshni et al. (2019) [46]	DenseNet-169+SVM	2862	2	X-ray	—-	0.8
H. Panwar et al. (2020) [50]	nCOVnet, transfer learning, deep CNN	337	2	X-ray	88.10%	0.881
M. Nour et al. (2020) [51]	Training CNN model, feature extraction, SVM	2,905	3	X-ray	98.97%,	0.994
P. Chhikara et al. (2020) [47]	Deep CNN with Transfer Learning	5866	2	X-ray	90.16%	0.911
M. A. Elaziz et al. (2020) [53]	MRFODE feature selection method, KNN classifier	1891	2	X-ray	96.09%	—-
		1560	2	X-ray	98.09%	—-

Therefore, building a classification method for various infections is one of the most critical issues prohibitively expensive for mass adoption. Deep transfer learning has been introduced for pneumonia detection from chest X-rays in different literature models. However, there is still a need for more improvements in the feature extraction and classification stages.

## III. Materials and Methods

This section introduces the chest X-ray datasets used in this paper and will also discuss the essential CNN deep transfer learning, multilayer perceptron neural network, and the original Squirrel Search (SS) optimization algorithm.

### A. Datasets

**FIGURE 1.**
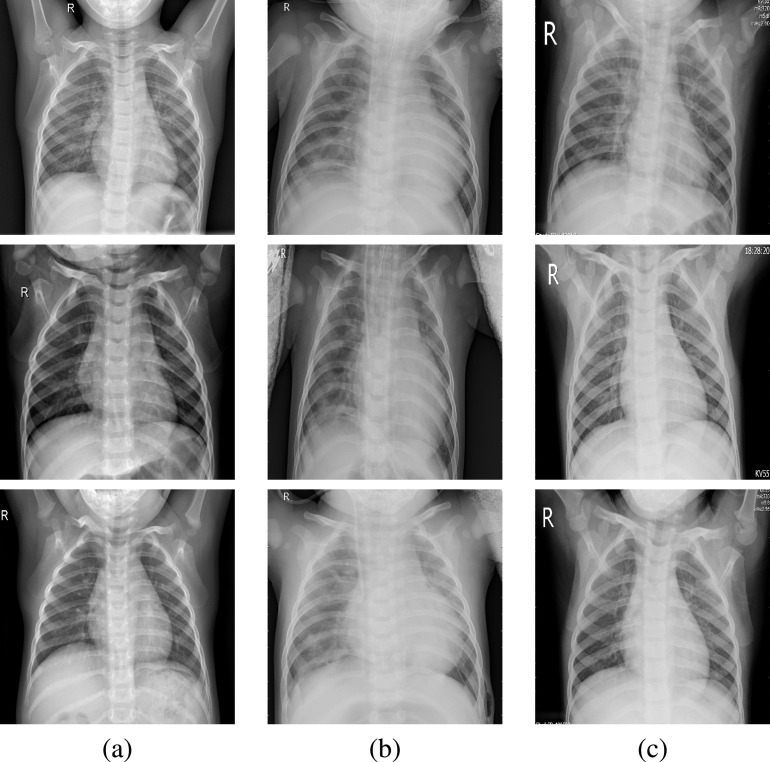
Samples of the original chest X-ray images [30]; (a) Normal cases, (b) Bacteria cases, and (c) Viral cases.

**FIGURE 2.**
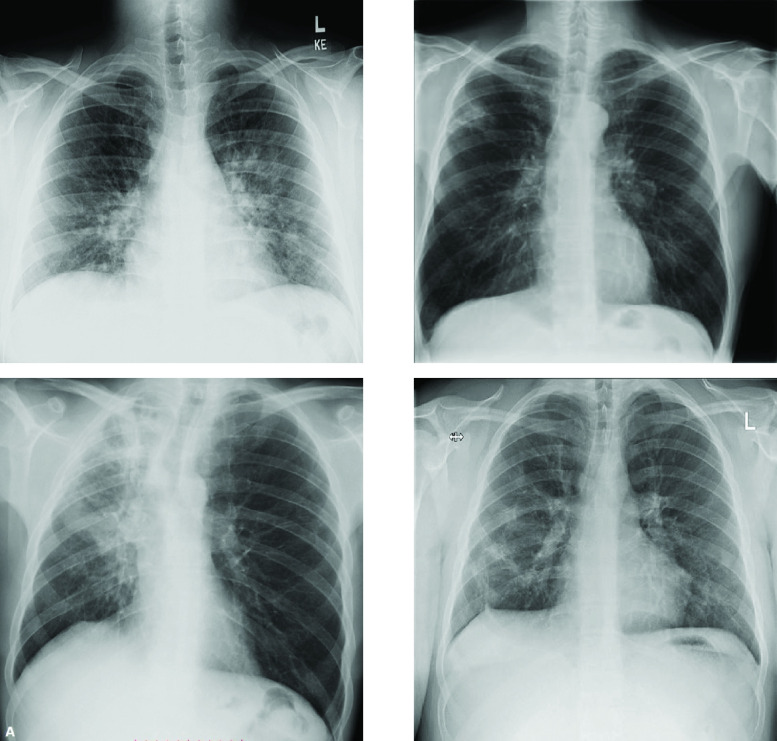
Samples of the original chest X-ray COVID-19 infected cases [31] tested in the experiments.

Chest (Pneumonia) X-ray images from Kaggle dataset [30] has been used. In (JPEG) format, the dataset has 5,863 X-rays. It is classified into two cases, either normal or pneumonia. In this paper, The Kaggle dataset has been selected because it is used in many forms of research globally and makes comparisons that can enrich scientific research. Figure 1(a) shows image samples of normal Pneumonia-free cases, while Fig. 1(b) and Fig. 1(c) present Pneumonia image samples (Bacteria and Viral cases), respectively. Another chest X-ray COVID-19 dataset [31] is also tested in the experiments and image samples are shown in Fig. 2. Besides indirect collection from hospitals and physicians, the COVID-19 dataset is obtained from public sources. In the GitHub repo, all data and images are released publicly. The tested dataset’s project was accepted by the Ethics Committee of the University of Montreal #CERSES-20-058-D.

### B. Deep Transfer Learning and CNN

In traditional learning, the model is isolated and based mainly on specific tasks and particular datasets [54]. The knowledge, in this learning, cannot be transferred from one model to another. In the transfer learning, knowledge, such as features and weights, can be transferred from the pre-trained model to new training models and different problems that may have fewer data. Transfer Learning is usually applied in various models for a dataset with less data than the dataset used to train the model. Multitask learning allows several tasks to learn simultaneously, which can help the model receive multiple tasks at once. The learner initially may have no idea about the target task [47].

CNN’s [55], a deep neural network, is known to be ideal for image processing applications and can achieve greater precision in the subject of disease classification than conventional approaches. It can thus be used in applications such as clustering, detecting objects, and classifying images. Several CNN models have recently been introduced, such as AlexNet, [56], VGGNet [57], GoogLeNet [58], Spotmole [59] and ResNet [60]. Convolution models used in the CNN models have different layers; higher classification accuracy is achieved if the number of convolution layers increases. [61]–[63].

Residual Network (ResNet) is known as an efficient CNN model [60]. The ResNet model was declared in 2016 to be the best paper at the Computer Vision and Pattern Recognition Conference (CVPR 06). The ResNet concept is based on the assumption that only a residual correction of the previous layer should be a deeper network training that can function efficiently, not transforming the whole feature space. The main idea of ResNets is not to learn the mapping from $x \rightarrow F(x)$, but instead learns the mapping from $x \rightarrow F(x) + G(x)$. Thus, if output $F(x)$, for input $x$, have the same dimension, $G(x) = x$ function is identity and the connection is identity. ResNet, without exploding and gradient vanishing issues, has much deeper neural network training.

### C. Multilayer Perceptron

**FIGURE 3.**
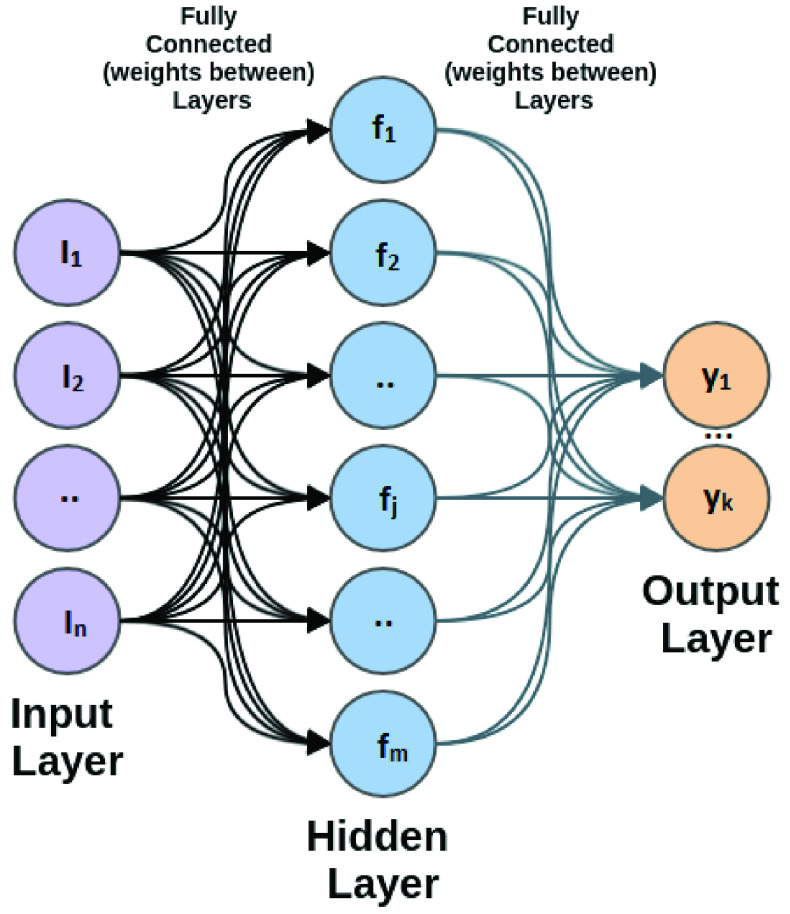
Neural Network - Multilayer Perceptron (MLP).

Feed-forward neural networks are considered supervised machine learning methods consisting of neurons distributed over fully connected layers. The first (input) layer maps the network input variables, and the last layer is the output one. Layers between the first and last layers are called hidden layers [64], [65]. Multilayer perceptron (MLP) is a common type of feed-forward network. The neurons interconnect in MLP, are one-directional fashion. The weights of the connections are within [−1, 1]. Figure 3 shows the MLP network, which includes one layer between input and output. To calculate the node output value, the weighted sum is firstly calculated as follows:

\begin{equation*} S_{j}= \sum _{i=1}^{n} w_{ij}I_{i} + \beta _{j}\tag{1}\end{equation*}

where $I_{i}$ represents input variable $i$ and $w_{ij}$ indicates connection weight between $I_{i}$ and neuron $j$ in the hidden layer. $\beta _{j}$ is bias value for this layer. By applying the sigmoid activation function, which is the mostly applied, node $j$ output is defined as

\begin{equation*} f_{j}(S_{j}) = \dfrac {1}{1+\exp ^{-S_{j}}}\tag{2}\end{equation*}

Based on the value of $f_{j}(S_{j})$ for all hidden layer neurons, the following equation can define the network output:

\begin{equation*} y_{k}= \sum _{j=1}^{m} w_{jk}f_{j}(S_{j}) + \beta _{k}\tag{3}\end{equation*}

where $w_{jk}$ indicates weights between neuron $j$ in the hidden layer and output node $k$ and $\beta _{k}$ is the bias value for the output layer.

### D. Squirrel Search Basic Optimization Algorithm

The Squirrel Search (SS) basic optimization algorithm simulates the search process of flying squirrels [32]. The SS algorithm considers that the squirrels are moving between three kinds of trees named normal, oak, and hickory trees. The oak and hickory trees are the nuts food source, while normal trees have no food source. Mathematically, the SS algorithm assumes the squirrels are flying to search for three oak trees and one hickory tree as nutritious food resources $N_{fs}$ available for $n$ flying squirrels ($FS$).

The flying agents’ locations is in matrix form as follows:

\begin{align*} FS = \begin{bmatrix} \displaystyle FS_{1,1} & FS_{1,2} & FS_{1,3} & {\dots }& FS_{1,d} \\ \displaystyle FS_{2,1} & FS_{2,2} & FS_{2,3} & {\dots }& FS_{2,d} \\ \displaystyle FS_{3,1} & FS_{3,2} & FS_{3,3} & {\dots }& FS_{3,d} \\ \displaystyle {\dots } & {\dots }& {\dots }& {\dots }& {\dots }\\ \displaystyle FS_{n,1} & FS_{n,2} & FS_{n,3} & {\dots }& FS_{n,d} \\ \displaystyle \end{bmatrix}\tag{4}\end{align*}

where $FS_{i,j}$ indicates $i^{th}$ flying squirrel in the $j^{th}$ dimension for $i \in {1,2,3, {\dots },n}$ and $j \in {1,2,3, {\dots },d}$. The initial locations of $FS_{i,j}$ are uniform distribution within lower and upper bounds. The fitness values $f={f_{1}, f_{2}, f_{3}, {\dots },f_{n}}$ are calculated for each flying squirrel as in the following array

\begin{align*} f = \begin{bmatrix} \displaystyle f_{1}(FS_{1,1}, FS_{1,2}, FS_{1,3}, {\dots },FS_{1,d}) \\ \displaystyle f_{2}(FS_{2,1}, FS_{2,2}, FS_{2,3}, {\dots },FS_{2,d}) \\ \displaystyle f_{3}(FS_{3,1}, FS_{3,2}, FS_{3,3}, {\dots },FS_{3,d}) \\ \displaystyle {\dots } \\ \displaystyle f_{n}(FS_{n,1}, FS_{n,2}, FS_{n,3}, {\dots },FS_{n,d}) \\ \displaystyle \end{bmatrix}\tag{5}\end{align*}

where the fitness value indicates the food source quality searched by each flying squirrel. The optimal value means a hickory tree. These values are then reordered in ascending order. The first best solution in declared to be $FS_{ht}$ on the hickory nut tree followed by three best solutions that are considered to be $FS_{at}$ on the acorn nuts trees. The remaining solutions are supposed to be $FS_{nt}$ on normal trees.

New location generation mathematically for each flying squirrel is considered as one of the three following cases:

Case 1: Location of $FS_{at}$ and moving to the hickory nut tree:
  \begin{align*} FS_{at}^{t+1} = \begin{cases} \displaystyle FS_{at}^{t} + d_{g} \times G_{c} (FS_{ht}^{t}-FS_{at}^{t}) & \text {if } R_{1} \geq P_{dp} \\ \displaystyle Random~~location & otherwise \end{cases}\tag{6}\end{align*}
Case 2: Location of $FS_{nt}$ and moving to the acorn nut trees:
  \begin{align*} FS_{nt}^{t+1} = \begin{cases} \displaystyle FS_{nt}^{t} + d_{g} \times G_{c} (FS_{at}^{t}-FS_{nt}^{t}) & \text {if } R_{2} \geq P_{dp} \\ \displaystyle Random~~location & otherwise \end{cases}\tag{7}\end{align*}
Case 3: Location of $FS_{nt}$ and moving to the hickory nut tree:
  \begin{align*} FS_{nt}^{t+1} = \begin{cases} \displaystyle FS_{nt}^{t} + d_{g} \times G_{c} (FS_{ht}^{t}-FS_{nt}^{t}) & \text {if } R_{3} \geq P_{dp} \\ \displaystyle Random~~location & otherwise \end{cases}\tag{8}\end{align*}
  where $R_{1}$, $R_{2}$, and $R_{3}$ are random numbers $\in [{0, 1}]$. The $d_{g}$ parameter is random distance for gliding and $t$ indicates the current iteration. $G_{c}$ is equal to 1.9 and it is constant to achieve exploration and exploitation balance, and the value of $P_{dp}$ probability is equal to 0.1 for the three cases.

The seasonal constant ($S_{c}$) is calculated from the following equation to check the monitoring condition ($S^{t}_{c} < S_{min}$) as

\begin{align*} S^{t}_{c}=&\sqrt {\sum _{k=1}^{d} (FS^{t}_{at,k} - FS_{ht,k})^{2}},\quad t = 1, 2, 3. \tag{9}\\ S_{min}=&\dfrac {10E^{-6}}{(365)^{t/(t_{m}/2.5)}}\tag{10}\end{align*}

where $t$ is the current iteration and $t_{m}$ represents the maximum iteration value. The value of $S_{min}$ can affect the algorithm exploration and exploitation capabilities during iterations. If specific condition is occurred, such flying squirrels’s relocation is modeled by Eq. 11

\begin{equation*} FS_{nt}^{new} = FS_{L} + Levy (n) \times (FS_{U}-FS_{L})\tag{11}\end{equation*}

where the distribution $Levy$ helps in encouraging better search space exploration. The calculation of the $Levy$ flight is as follows:

\begin{equation*} Levy (x) = 0.01 \times \frac {r_{a} \times \sigma }{|r_{b}|^{\frac {1}{\beta }}}\tag{12}\end{equation*}

where the parameters $r_{a}$ and $r_{b}$ are random in [0, 1]. $\beta $ is equal to 1.5 in the SS algorithm and $\sigma $ is calculated as

\begin{equation*} \sigma = \left ({\dfrac {\Gamma (1+\beta) \times \sin \left({\frac {\pi \beta }{2}}\right)}{\Gamma \left({\frac {1 + \beta }{2}}\right) \times \beta \times 2^{\left({\frac {\beta -1}{2}}\right)}} }\right) ^{1 / \beta }\tag{13}\end{equation*}

where $\Gamma (x) = (x-1)!$. The basic Squirrel Search (SS) optimization algorithm is explained step by step in Algorithm 1.

Algorithm 1 **Basic SS Optimization Algorithm [32]**
1: **Initialize** SS population $FS_{i} (i = 1, 2, \ldots, n)$ with size $n$ using Eq. (4), maximum iterations $t_{m}$, and fitness function $F_{n}$.
2: **Initialize** SS parameters $R_{1}$, $R_{2}$, $R_{3}$, $n_{1}$, $n_{2}$, $n_{3}$, $P_{dp}$, $G_{c}$, $t = 1$
3: **Calculate** fitness function $F_{n}$ for each $FS_{i}$ using Eq. (5)
4: **Sort** flying squirrels locations in ascending order
5: **Find** the first best individual $FS_{ht}$
6: **Find** the next three best individuals $FS_{at}$
7: **Find** the normal individuals $FS_{nt}$
8: **while** $t \leq t_{m}$ (Stopping condition) **do**
9: **for** ($t = 1: n_{1}$) **do**
10: **if** ($R_{1} \geq P_{dp}$) **then**
11: $FS_{at}^{t+1} = FS_{at}^{t} + d_{g} \times G_{c} (FS_{ht}^{t}-FS_{at}^{t})$
12: **else**
13: $FS_{at}^{t+1} = Random\,\,\,\,location$
14: **end if**
15: **end for**
16: **for** ($t = 1: n_{2}$) **do**
17: **if** ($R_{2} \geq P_{dp}$) **then**
18: $FS_{nt}^{t+1} = FS_{nt}^{t} + d_{g} \times G_{c} (FS_{at}^{t}-FS_{nt}^{t})$
19: **else**
20: $FS_{nt}^{t+1} = Random\,\,\,\,location$
21: **end if**
22: **end for**
23: **for** ($t = 1: n_{3}$) **do**
24: **if** ($R_{3} \geq P_{dp}$) **then**
25: $FS_{nt}^{t+1} = FS_{nt}^{t} + d_{g} \times G_{c} (FS_{ht}^{t}-FS_{nt}^{t})$
26: **else**
27: $FS_{nt}^{t+1}= Random\,\,\,\,location$
28: **end if**
29: **end for**
30: **Calculate** seasonal constant ($S^{t}_{c}$) using Eq. (9)
31: **Calculate** minimum value of seasonal constant ($S_{min}$) using Eq. (10)
32: **if** ($S^{t}_{c} < S_{min}$) **then**
33: $FS_{nt}^{new} = FS_{L} + Levy (n) \times (FS_{U}-FS_{L})$
34: **end if**
35: **Update** $S_{min}$ using Eq. (10)
36: **Set** $t = t + 1$
37: **end while**
38: **Return** optimal solution $FS_{ht}$

## IV. Proposed Classification Method

The proposed classification method consists of two stages. The first stage has a feature engineering process, including image augmentation, CNN training using the ResNet-50 model, transfer learning, and dropout. The proposed ASSOA algorithm is then applied to select features from the ResNet-50 model’s extracted features. The second stage involves the classification process to classify cases in which the MLP is optimized by the proposed ASSOA algorithm (ASSOA + MLP).

### A. Feature Engineering Stage

The ResNet-50 model is applied in this stage as a part of the proposed method for features extraction from the chest X-ray images in the fully connected layer by altering the nodes and doing a fine-tuning based on the input dataset. Each input image is resized to $224 \times 224$ pixels to be suitable for the model. Then, the Min-Max-Scalar is used to normalize the $i$th input image $x_{i}$ to a scale from 0 to 1 by applying the following equation.

\begin{equation*} x'_{i}=\dfrac {x_{i} - min(x_{i})}{max(x_{i}) - min(x_{i})}\tag{14}\end{equation*}

After the resizing and normalization, the output image $x'_{i}$ is used as input to the CNN model. The adopted CNN structure of the number of filters and layers and the related specifications are identical to the ResNet-50 model. This model focuses on classifying input case categories. To reduce the overfitting problem during network learning, two regularization techniques of dropout and image augmentation have been applied in this research. The dropout is applied during the training procedure of CNN, and image augmentation [66] is used for the X-ray images’ input images. Data Augmentation is applied to improve the quality and size of the training datasets.

### B. The Proposed ASSOA Algorithm

**FIGURE 4.**
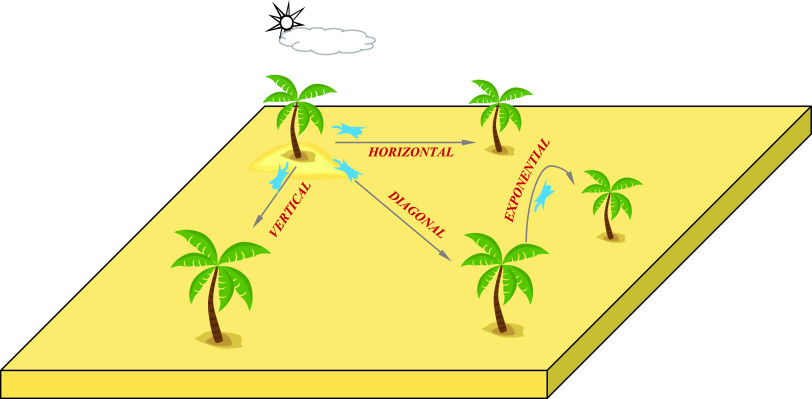
Squirrel movements in the Proposed Advanced Squirrel Search Optimization Algorithm (ASSOA).

The proposed ASSOA algorithm adds horizontal, vertical, diagonal, and exponential movements to the basic moves in the search process of flying squirrels, as shown in Fig. 4. The ASSOA algorithm considers, as in the basic SS algorithm, that the squirrels are moving between three kinds of trees named normal, oak, and hickory trees. The nuts food sources are the oak and hickory trees, while there are no food sources on the other trees.

Mathematically, the ASSOA algorithm assumes the squirrels are flying in directions shown in Fig. 4 to search for one hickory tree, the best solution, and three oak trees, next best solutions, as nutritious food resources $N_{fs}$ available for $n$ flying squirrels ($FS$). The following matrices represent the flying squirrels’ locations and velocities:

\begin{align*} FS=&\begin{bmatrix} \displaystyle FS_{1,1} & FS_{1,2} & FS_{1,3} & {\dots }& FS_{1,d} \\ \displaystyle FS_{2,1} & FS_{2,2} & FS_{2,3} & {\dots }& FS_{2,d} \\ \displaystyle FS_{3,1} & FS_{3,2} & FS_{3,3} & {\dots }& FS_{3,d} \\ \displaystyle {\dots } & {\dots }& {\dots }& {\dots }& {\dots }\\ \displaystyle FS_{n,1} & FS_{n,2} & FS_{n,3} & {\dots }& FS_{n,d} \\ \displaystyle \end{bmatrix} \tag{15}\\ V=&\begin{bmatrix} \displaystyle V_{1,1} & V_{1,2} & V_{1,3} & {\dots }& V_{1,d} \\ \displaystyle V_{2,1} & V_{2,2} & V_{2,3} & {\dots }& V_{2,d} \\ \displaystyle V_{3,1} & V_{3,2} & V_{3,3} & {\dots }& V_{3,d} \\ \displaystyle {\dots } & {\dots }& {\dots }& {\dots }& {\dots }\\ \displaystyle V_{n,1} & V_{n,2} & V_{n,3} & {\dots }& V_{n,d} \\ \displaystyle \end{bmatrix}\tag{16}\end{align*}

where $FS_{i,j}$ indicates $i^{th}$ flying squirrel location in the $j^{th}$ dimension for $i \in {1,2,3, {\dots },n}$ and $j \in {1,2,3, {\dots },d}$. $V_{i,j}$ indicates $i^{th}$ flying squirrel velocity in the $j^{th}$ dimension for $i \in {1,2,3, {\dots },n}$ and $j \in {1,2,3, {\dots },d}$. The initial locations of $FS_{i,j}$ are uniform distribution within lower and upper bounds. The fitness values $f={f_{1}, f_{2}, f_{3}, {\dots },f_{n}}$ are calculated for each flying squirrel as in Eq. 5. The optimal value means a hickory tree. These values are then sorted in ascending order. The first best solution in declared to be $FS_{ht}$ on the hickory nut tree followed by three best solutions that are considered to be $FS_{at}$ on the acorn nuts trees. The remaining solutions are supposed to be $FS_{nt}$ on normal trees.

In the ASSOA algorithm, the new location generation for each flying squirrel is updated as in the following cases. For a random value $p$, the following cases will be applied if $p \geq 0.5$:

Case 1: Location of $FS_{at}$ and moving to the hickory nut tree:
  \begin{align*} FS_{at}^{t+1} \!=\! \begin{cases} \displaystyle FS_{at}^{t} \!+ \!d_{g} \times G_{c} (FS_{ht}^{t}-FS_{at}^{t}) & \text {if } R_{1} \geq P_{dp} \\ \displaystyle Random~location & otherwise \end{cases}\tag{17}\end{align*}
Case 2: Location of $FS_{nt}$ and moving to the acorn nut trees:
  \begin{align*} FS_{nt}^{t+1} \!=\! \begin{cases} \displaystyle FS_{nt}^{t} \!+ \!d_{g} \times G_{c} (FS_{at}^{t}-FS_{nt}^{t}) & \text {if } R_{2} \geq P_{dp} \\ \displaystyle Random~~location & otherwise \end{cases}\tag{18}\end{align*}
Case 3: Location of $FS_{nt}$ and moving to the hickory nut tree:
  \begin{align*} FS_{nt}^{t+1}\! = \!\begin{cases} \displaystyle FS_{nt}^{t} \!+\! d_{g} \times G_{c} (FS_{ht}^{t}-FS_{nt}^{t}) & \text {if } R_{3} \geq P_{dp} \\ \displaystyle Random~~location & otherwise \end{cases}\tag{19}\end{align*}
  where $R_{1}$, $R_{2}$, and $R_{3}$ are random numbers $\in [{0, 1}]$. The $d_{g}$ parameter is random distance for gliding and $t$ indicates the current iteration. $G_{c}$ is equal to 1.9 and it is constant to achieve the exploration and exploitation balance, and the value of $P_{dp}$ probability is equal to 0.1 for the three cases.
  For the random value $p$, the following cases will be applied if $p < 0.5$:
Case 4: Location of $FS_{nt}$ and moving diagonally:
  \begin{align*} FS_{nt}^{t+1} = \begin{cases} \displaystyle FS_{nt}^{t} + V_{nt}^{t} + \\ \displaystyle c_{1}~r (FS_{ht}^{t}-FS_{nt}^{t}) + \\ \displaystyle c_{2}~r (FS_{at}^{t}-FS_{nt}^{t}) & \text {if } P_{a} < a \\ \displaystyle Random~~FS^{t}_{rand} \in FS^{t}_{nt} & otherwise \end{cases}\tag{20}\end{align*}
  where $c_{1}$, $c_{2}$, $r$, $P_{a}$, and $a$ are random numbers $\in [{0, 1}]$. In case of choosing a random agent $FS^{t}_{rand}$ from the normal agents $FS^{t}_{nt}$, the fitness value $F_{n}(FS^{t}_{rand})$ for $FS^{t}_{rand}$ and $F_{n}(FS_{nt}^{t}$ for $FS^{t}_{nt}$ will be calculated to decide about the horizontal and vertical movement. In case of $F_{n}(FS^{t}_{rand}) < F_{n}(FS_{nt}^{t})$, the movement will be vertically and it will be horizontally otherwise as follow
Case 5: Location of $FS_{nt}$ and moving vertically or horizontally based on the fitness value $F_{n}(FS^{t}_{rand})$:
  \begin{align*} FS_{nt}^{t+1} = \begin{cases} \displaystyle FS_{nt}^{t} + V_{nt}^{t} + \\ \displaystyle c_{3}~r (FS^{t}_{rand}- FS_{nt}^{t}) & \text {if } F_{n}(FS^{t}_{rand}) \\ \displaystyle & < F_{n}(FS_{nt}^{t}) \\ \displaystyle FS_{nt}^{t} + V_{nt}^{t} + \\ \displaystyle c_{1}~r (FS_{ht}^{t}-FS_{nt}^{t}) & otherwise \end{cases}\tag{21}\end{align*}
  where $c_{3}$ is a random number $\in [{0, 1}]$. The last case will be applied if the condition of the horizontal and vertical movement is not achieved.
Case 6: Location of $FS_{nt}$ and moving will be exponentially:
  \begin{equation*} FS_{nt}^{t+1} = FS_{nt}^{t} + |(FS^{t}_{rand}-FS_{nt}^{t})|\exp (bt)\cos (2 \pi t)\tag{22}\end{equation*}
  where $b$ is a random number $\in [{0, 1}]$.

**FIGURE 5.**
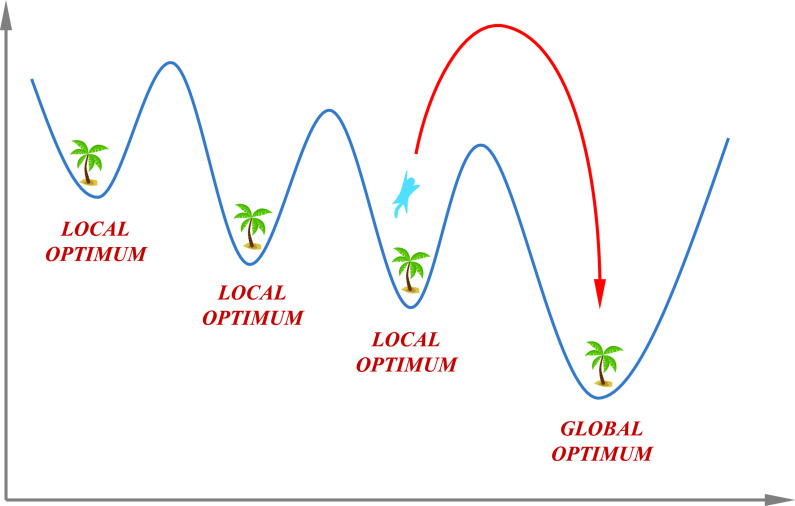
Local and global optima of the proposed ASSOA algorithm.

The seasonal constant ($S_{c}$) and the minimal value of the seasonal constant $S_{min}$ are calculated from Eq. 9 and Eq. 10 to check the monitoring condition ($S^{t}_{c} < S_{min}$) for $t$ is the current iteration and $t_{m}$ indicates iterations maximum value. The value of $S_{min}$ can affect the algorithm exploration and exploitation capabilities during iterations. If specific condition is occurred, such flying squirrels’s relocation is modeled by Eq. 23 which has the effect on local and global optima as shown in Fig. 5:

\begin{align*} FS_{nt}^{new} = FS_{ht}^{t} + 2 r \left({(FS_{ht}^{t} - FS_{nt}^{t})\left({1-\left({\frac {FS_{ht}^{t} + FS_{nt}^{t}}{FS_{nt}^{t}}}\right)^{2}}\right)}\right) \\\tag{23}\end{align*}

The proposed ASSOA algorithm is explained step by step in Algorithm 2. The proposed algorithm’s computational complexity will be discussed as shown in Algorithm 2. Let the number of population be $n=n_{1}+n_{2}+n_{3}$; the maximum number of iterations be $t_{m}$. For parts of the ASSOA algorithm, the time complexity will be defined as in the following points:

• Initialize of ASSOA population: $O$ (1).
• Initialize of ASSOA parameters $R_{1}$, $R_{2}$, $R_{3}$, $n_{1}$, $n_{2}$, $n_{3}$, $P_{dp}$, $G_{c}$, $c_{1}$, $c_{2}$, $c_{3}$, $r$, $b$, $P_{a}$, $P_{d}$, $a$, $d$, $p$: $O$ (1).
• Calculate fitness function for each agent: $O$ ($n$).
• Sorting agents in ascending order: $O$ ($n$).
• Finding first best individual, next three best individuals, normal individuals: $O$ ($n$).
• Positions’ updating for each agent in case 1: $O$ ($t_{m} \times n_{1}$).
• Positions’ updating for each agent in case 2: $O$ ($t_{m} \times n_{2}$).
• Positions’ updating for each agent in case 3: $O$ ($t_{m} \times n_{3}$).
• Positions’ updating for each agent in case 4: $O$ ($t_{m}$).
• Positions’ updating for each agent in case 5: $O$ ($t_{m}$).
• Positions’ updating for each agent in case 6: $O$ ($t_{m}$).
• Calculating the seasonal constant: $O$ ($t_{m}$).
• Calculating the minimum value of seasonal constant: $O$ ($t_{m}$).
• Relocation of agents: $O$ ($t_{m}$).
• 3 Iteration number increment: $O$ ($t_{m}$).
• Returning the best individual: $O$ (1)

The above analysis indicates that the proposed ASSOA algorithm’ complexity of computations is $O$ ($t_{m} \times n$) and in case of a problem with $d$ dimension is $O$ ($t_{m} \times n \times d$).

Algorithm 2 **Proposed ASSOA Algorithm**
1: **Initialize** ASSOA population $FS_{i} (i = 1, 2, \ldots, n)$ with size $n$ using Eq. (15), velocities $V_{i} (i = 1, 2, \ldots, n)$ using Eq. (16), maximum iterations $t_{m}$, and fitness function $F_{n}$.
2: **Initialize** ASSOA parameters $R_{1}$, $R_{2}$, $R_{3}$, $n_{1}$, $n_{2}$, $n_{3}$, $P_{dp}$, $G_{c}$, $c_{1}$, $c_{2}$, $c_{3}$, $r$, $b$, $P_{a}$, $P_{d}$, $a$, $d$, $p$, $t = 1$
3: **Calculate** fitness function $F_{n}$ for each $FS_{i}$ using Eq. (5) and Sort flying squirrels locations in ascending order
4: **Find** the first best individual $FS_{ht}$, the next three best individuals $FS_{at}$, the normal individuals $FS_{nt}$
5: **while** $t \leq t_{m}$ (Stopping condition) **do**
6: **if** ($p \geq 0.5$) **then**
7: **for** ($t = 1: n_{1}$) **do**
8: **if** ($R_{1} \geq P_{dp}$) **then**
9: $FS_{at}^{t+1} = FS_{at}^{t} + d_{g} \times G_{c} (FS_{ht}^{t}-FS_{at}^{t})$
10: **else**
11: $FS_{at}^{t+1} = Random\,\,\,\,location$
12: **end if**
13: **end for**
14: **for** ($t = 1: n_{2}$) **do**
15: **if** ($R_{2} \geq P_{dp}$) **then**
16: $FS_{nt}^{t+1} = FS_{nt}^{t} + d_{g} \times G_{c} (FS_{at}^{t}-FS_{nt}^{t})$
17: **else**
18: $FS_{nt}^{t+1} = Random\,\,\,\,location$
19: **end if**
20: **end for**
21: **for** ($t = 1: n_{3}$) **do**
22: **if** ($R_{3} \geq P_{dp}$) **then**
23: $FS_{nt}^{t+1} = FS_{nt}^{t} + d_{g} \times G_{c} (FS_{ht}^{t}-FS_{nt}^{t})$
24: **else**
25: $FS_{nt}^{t+1}= Random\,\,\,\,location$
26: **end if**
27: **end for**
28: **else**
29: **if** ($P_{a} < a$) **then**
30: $FS_{nt}^{t+1} = FS_{nt}^{t} + V_{nt}^{t} + c_{1}\,\,r (FS_{ht}^{t}-FS_{nt}^{t}) + c_{2}\,\,r (FS_{at}^{t}-FS_{nt}^{t})$
31: **else**
32: **Choose** random agent $FS^{t}_{rand}$ from normal agents $FS^{t}_{nt}$
33: **if** ($P_{d} < d$) **then**
34: **Calculate** fitness function $F_{n}(FS^{t}_{rand})$ for $FS^{t}_{rand}$
35: **if** ($F_{n}(FS^{t}_{rand}) < F_{n}(FS_{nt}^{t})$) **then**
36: $FS_{nt}^{t+1} = FS_{nt}^{t} + V_{nt}^{t} + c_{3}\,\,r (FS^{t}_{rand}-FS_{nt}^{t})$
37: **else**
38: $FS_{nt}^{t+1} = FS_{nt}^{t} + V_{nt}^{t} + c_{1}\,\,r (FS_{ht}^{t}-FS_{nt}^{t})$
39: **end if**
40: **else**
41: $FS_{nt}^{t+1} = FS_{nt}^{t} + |(FS^{t}_{rand}-FS_{nt}^{t})|\exp (bt)\cos (2 \pi t)$
42: **end if**
43: **end if**
44: **end if**
45: **Calculate** seasonal constant ($S^{t}_{c}$) using Eq. (9)
46: **Calculate** minimum value of seasonal constant ($S_{min}$) using Eq. (10)
47: **if** ($S^{t}_{c} < S_{min}$) **then**
48: $FS_{nt}^{new} = FS_{ht}^{t} + 2\,\,r \left({(FS_{ht}^{t} - FS_{nt}^{t})\left({1-\left({\frac {FS_{ht}^{t}+FS_{nt}^{t}}{FS_{nt}^{t}}}\right)^{2}}\right)}\right)$
49: **end if**
50: **Update** $S_{min}$ using Eq. (10)
51: **Set** t = t +1
52: **end while**
53: **Return** optimal solution $FS_{ht}$

### C. Binary Optimizer

In the feature selection problem, the search space is represented by only binary values of 0 and 1. Thus, the proposed ASSOA algorithm’s continuous values are converted into binary values for the process of feature selection based on the ResNet-50 model’s extracted features. The following equation will be applied to get the binary values from the standard continuous values of the proposed ASSOA algorithm.

\begin{align*} FS_{d}^{(t+1)}=&\begin{cases} \displaystyle 1 & \text {if } Sigmoid(x) \geq 0.5 \\ \displaystyle 0 & otherwise, \end{cases} \\ Sigmoid(x)=&\dfrac {1}{1+\exp ^{-10(x-0.5)}},\tag{24}\end{align*}

where $FS_{d}^{(t+1)}$ is the binary position at iteration $t$ of $d$ dimension. The $Sigmoid$ function scales the continuous values to be zero or one. $Sigmoid(x) \geq 0.5$ condition is employed here to filter the values to be o or 1. the $x$ value indicates the best solution of the algorithm which is denoted as $FS_{ht}$ in Algorithm 2.

### D. Fitness Function

The fitness function measures the optimizer solutions’ quality. The function is dependent on the classification error rate and the selected features. The excellent solution corresponds to a set of features that give lower features and classification error rate. To evaluate the solution quality, Eq. 25 can be employed

\begin{equation*} F_{n} = h_{1} Err(O)+ h_{2} \dfrac {|s|}{| f|}\tag{25}\end{equation*}

where $Err(O)$ indicates the optimizer error rate, $s$ denotes the set of features selected by the optimizer, $f$ denotes the features’ total number. The $h_{1} \in [{0,1}], h_{2}= 1- h_{1}$ values manage the importance of the error rate of classification process and the selected feature number.

## V. Experimental Results

There are three scenarios in the experiments. The first scenario shows the effectiveness of four CNN models for classifying the chest X-ray cases and offers the importance of features extraction for the next stage. The second scenario is designed to test and compare the proposed ASSOA algorithm to other optimization algorithms for feature selection. The third scenario is conducted to test the proposed ASSOA algorithm’s ability as a classifier for improving the classification accuracy based on MLP. Wilcoxon’s rank-sum test is performed to verify the proposed algorithm’s superiority statistically. For the chest X-ray datasets, the images are separated randomly into training images of (60%), validation images of (20%), testing images of (20%). The data in the training process is used to train the CNN model. In contrast, the validation process data is applied for verification purposes, and the testing data evaluated the efficiency of the proposed method for the unknown chest X-ray cases.

### A. First Scenario

The classification accuracy of the four CNN models namely AlexNet [56], VGGNet [57], GoogLeNet [58], and ResNet-50 [60] is claculated in this scenario for the tested chest X-ray dataset. Let $TP$ indicates true-positive value, $FP$ represents false-positive value, $TN$ indicates true-negative value, and $FN$ represents false-negative value. The performance metrics, such as accuracy, precision, and F-score [29], are calculated to measure the classification performance of the CNN models as shown in Table 2. The results of this scenario including the required CPU time are shown in Table 3.

**TABLE 2:** Performance Metrics for Classification

Metric	Value
Accuracy	$\dfrac {TP + TN}{TP + TN + FP + FN}$
Specificity	$\dfrac {TN}{TN + FP}$
Sensitivity	$\dfrac {TP}{TP + FN}$
Negative Predictive Value (NPV)	$\dfrac {TN}{TN + FN}$
Precision (PPV)	$\dfrac {TP}{TP + FP}$
F-score	$2 \times \dfrac {PPV \times TPR}{PPV + TPR}$

**TABLE 3:** Performance Metrics Outputs of the Compared Deep Learning Approaches

Method/Metric	Accuracy	Sensitivity	Specificity	PPV	NPV	F-score	Time (s)
AlexNet	0.820	0.840	0.610	0.670	0.850	0.745	724
VGGNet	0.869	0.741	0.751	0.997	0.249	0.850	747
GoogLeNet	0.880	0.910	0.890	0.800	0.920	0.851	841
ResNet-50	0.910	0.950	0.920	0.840	0.950	0.892	203

Table 4 presents the settings of the CNN experimental setup in this scenario. The default parameters are used in this case since the current stage is employed for feature extraction of the chest X-ray images from the CNN model to be used for the next scenario. The highest accuracy achieved in this case, for the X-ray images, is (91.0%) by the ResNet-50 model with an F-score of (89.2%) and required time of (203) seconds. According to the promising performance of the ResNet-50 model, a set of features is extracted from the model’s earlier layers since the model accuracy should be improved for the critical cases. In the second scenario, these features are employed to extract the best classification features by the proposed ASSOA algorithm.

**TABLE 4:** CNN Experimental Setup

Parameter	Value
CNN training options (Default)	
RateDropFactor	0.1000
Momentum Learn	0.9000
L2Regularization	1.0000 e-04
LearnRateDropPeriod	10
GradientThreshold	Inf
GradientThresholdMethod	12 norm
ValidationData	imds
VerboseFrequency	50
ValidationPatience	Inf
ValidationFrequency	50
ResetInputNormalization	1
CNN training options (Custom)	
InitialLearnRate	1.0000 e-04
ExecutionEnvironment	gpu
MiniBatchSize	8
MaxEpochs	20
Verbose	0
Shuffle	every-epoch
LearnRateSchedule	piecwise
Optimizer	sgdm

### B. Second Scenario

In this scenario, the efficiency of feature selection by the proposed ASSOA algorithm is investigated. ASSOA algorithm performance is compared with the basic Squirrel Search (SS) optimization algorithm [32], Grey Wolf Optimizer (GWO) [29], and Genetic Algorithm (GA) [33] based on performance metrics shown in Table 5. Let $M$ be the number of runs of an optimizer; $g_{j}^{*}$ represents the best solution at the run number $j$; $size(g_{j}^{*})$ is the size of the vector $g_{j}^{*}$. $N$ is the number of tested points; $C_{i}$ is the classifier’s output label for a point $i$; $L_{i}$ is the class’s label for a point $i$; the total number of features ($D$); and matching between two inputs is calculated by $Match$ function. The metrics used in this scenario are average error, select size, fitness, best and worst fitness, and standard deviation fitness. ASSOA algorithm configuration setting is shown in Table 6. $h_{1}$ parameter in the objective function is assigned to 0.99 and $h_{2}$ parameter to 0.01. The configuration of the SS, GWO, GA algorithms, including the number of iterations, agents, and parameters, is shown in Table 7.

**TABLE 5:** Performance Metrics for Feature Selection

Metric	Value
Average Error	$1- \frac {1}{M} \sum _{j=1}^{M} \frac {1}{N} \sum _{i=1}^{N} Match(C_{i}, L_{i}) $
Average Select Size	$\frac {1}{M} \sum _{j=1}^{M} \dfrac {size(g_{j}^{*})}{D} $
Average Fitness	$\frac {1}{M} \sum _{j=1}^{M} g_{j}^{*}$
Best Fitness	$Min_{j=1}^{M} g_{j}^{*} $
Worst Fitness	$Max_{j=1}^{M} g_{j}^{*} $
Standard Deviation	$\sqrt {\frac {1}{M-1} \sum (g_{j}^{*} - Mean)^{2} }$

**TABLE 6:** Proposed ASSOA Algorithm Configuration

Parameter	Value
Number of iterations	50
Number of agents	10
$R_{1}$	[0,1]
$R_{2}$	[0,1]
$R_{3}$	[0,1]
$G_{c}$	1.9
$P_{dp}$	0.1
$c_{1}$,$c_{2}$,$c_{3}$	[0,1]
$P_{a}$	[0,1]
$P_{d}$	[0,1]
$a$, $r$, $b$, $p$	[0,1]

**TABLE 7:** Compared Algorithms Configuration

Algorithm	Parameter (s)	Value (s)
SS	Number of iterations	50
	Number of agents	10
	$R_{1}$	[0,1]
	$R_{2}$	[0,1]
	$R_{3}$	[0,1]
	$G_{c}$	1.9
	$P_{dp}$	0.1
GWO	No. of iterations	50
	No. of wolves	10
	$a$	2 to 0
GA	Generations	50
	Population size	10
	Mutation ratio	0.1
	Crossover	0.9
	Selection mechanism	Roulette wheel

**FIGURE 6.**
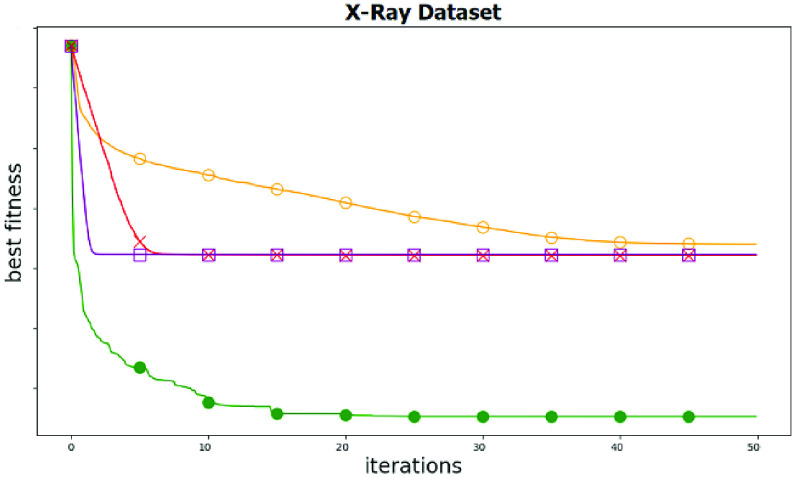
Proposed ASSOA Convergence curves compared to other techniques; Green, purple, red, and yellow lines indicates ASSOA, SS, GWO, GA algorithms, respectively.

Table 8 shows the ASSOA, SS, GWO, and GA algorithms’ output results in this scenario. For the displayed results, if the optimizer can select the proper set of features, the error is minimized. ASSOA can achieve a minimum average error of (0.2113) for feature selection. Based on the tested problem’s minimum error, the ASSOA algorithm is the best, and GA is the worst. This means that the proposed ASSOA algorithm achieved better results than the original SS algorithm. In terms of standard deviation, the ASSOA algorithm has the lowest value than other algorithms that indicate the algorithm’s robustness and stability. Figure 6 shows the ASSOA convergence curve compared to different algorithms. The figure demonstrates the optimizer exploitation capability and the ability of the algorithm to avoid local optima. The figure results show the reliability and robustness of the ASSOA algorithm to get the optimal set of features in a minimum time.

**TABLE 8:** Performance of the Proposed ASSOA Algorithm for Feature Selection Compared to Other Algorithms

Metric / Optimizer	ASSOA	SS	GWO	GA
Average error	0.2113	0.2956	0.3243	0.3317
Average Select size	0.7333	0.7967	0.8000	0.8000
Average Fitness	0.1236	0.1602	0.1673	0.1752
Best Fitness	1.2812	1.3012	1.3909	1.4009
Worst Fitness	1.4061	1.4854	1.5057	1.5142
Standard deviation Fitness	0.0047	0.0055	0.0061	0.0067

The p-values of the ASSOA algorithm are tested compared to SS, GWO, and GA algorithms by Wilcoxon’s rank-sum test. The employed test can get if there is a significant difference between the ASSOA algorithm and other algorithms. If the p-value < 0.05, this indicates that the ASSOA algorithm results are significantly different from other algorithms. If p-value >0.05, this indicates that the algorithm results have no significant difference. The p-value results in this scenario are shown in Table 9. Results show that the p-values are less than 0.05, proving the superiority of the proposed ASSOA algorithm and that the algorithm has statistically significant.

**TABLE 9:** Proposed ASSOA Algorithm’s p-Values in Comparison to Other Algorithms Using Wilcoxon’s Rank-Sum Based on Average Error Metric

Compared Algorithms	SS	GWO	GA
p-value	1.21E-05	1.21E-05	1.21E-05

### C. Third Scenario

The last scenario checks the classification accuracy of the ASSOA algorithm based on MLP (ASSOA + MLP) in comparison with other algorithms of SS + MLP, GWO + MLP, and GA + MLP. The classification performance is tested for chest X-ray cases and other cases based on chest X-ray COVID-19. The configuration of the proposed ASSOA algorithm and the compared algorithms are shown in Table 6 and Table 7, respectively. Metrics of the classification performance used in this scenario are presented in Table 2.

#### 1) Chest X-Ray Classification Results

**FIGURE 7.**
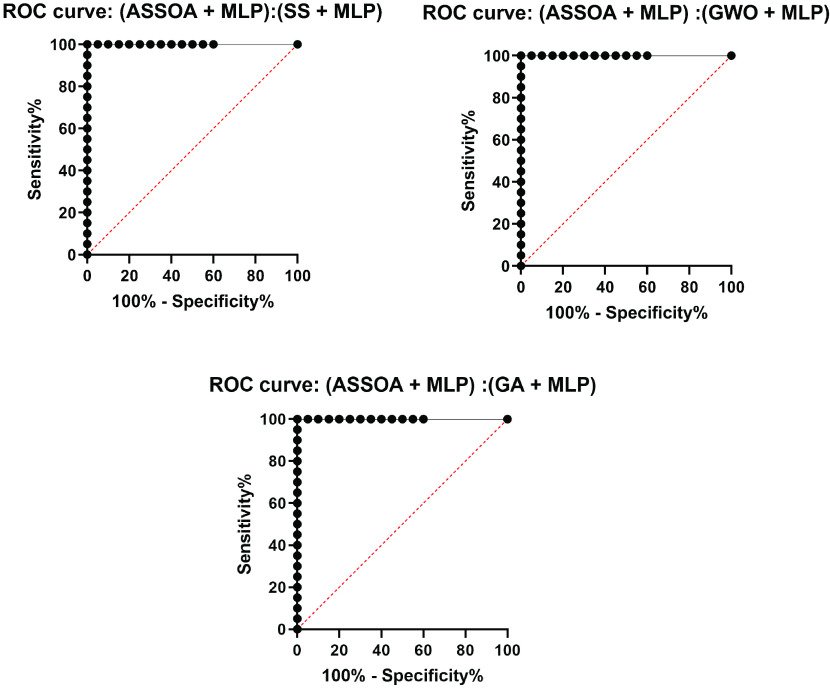
ROC curves for ASSOA algorithm versus compared algorithms.

**FIGURE 8.**
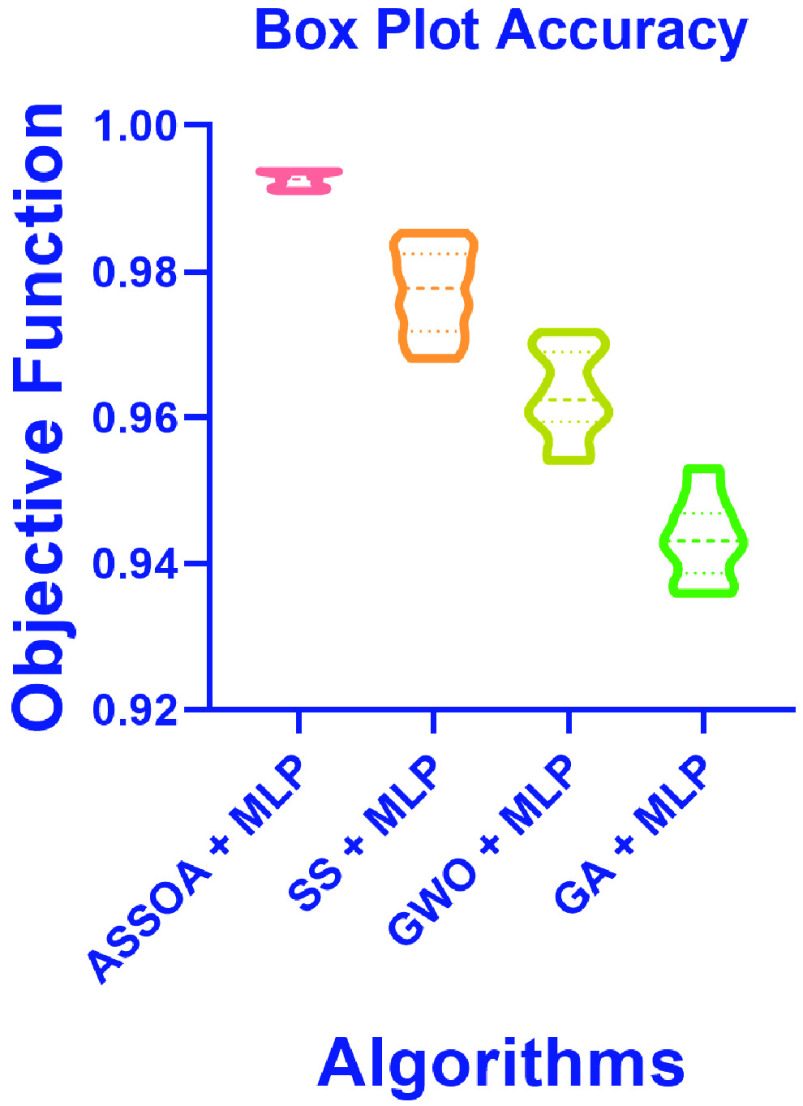
Box plot accuracy of the proposed ASSOA + MLP algorithm compared to other algorithms for chest X-ray dataset.

**FIGURE 9.**
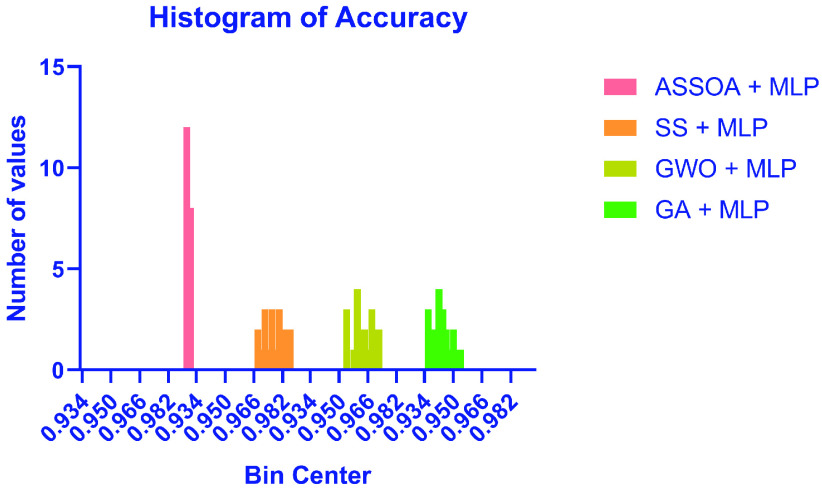
Histogram of accuracy of the proposed ASSOA + MLP algorithm compared to other algorithms for chest X-ray dataset.

The results of the ASSOA + MLP algorithm and other algorithms regarding accuracy are shown in Table 10. The proposed algorithm (ASSOA + MLP) from the descriptive statistics, as shown in Table 10, can achieve a mean accuracy of (99.26%) and a standard deviation of (0.001098) within (135) seconds to classify a new input X-ray chest image which outperforms other algorithms. The ROC curves of the proposed ASSOA algorithm based on MLP versus the compared classification algorithms are shown in Figure 7. From this figure, the proposed algorithm can highly distinguish among the X-ray chest images with a high AUC value equal to (0.9875). The Box plot accuracy and Histogram of accuracy are also tested, and the output figures are shown in Figures 8 and 9. These figures show the stability and consistency of the proposed algorithm for the classification of different cases.

**TABLE 10:** Descriptive Statistics and the Classification Accuracy of the ASSOA Algorithm Based on MLP in Comparison to Other Algorithms for Chest X-Ray Images

	ASSOA + MLP	SS + MLP	GWO + MLP	GA + MLP
Number of values	20	20	20	20
Minimum (Accuracy)	0.991	0.9681	0.9541	0.9359
25% Percentile	0.9914	0.9718	0.9594	0.9387
Median (Accuracy)	0.9926	0.9777	0.9624	0.9431
75% Percentile	0.9938	0.9825	0.969	0.9469
Maximum (Accuracy)	0.9938	0.9853	0.9718	0.9531
Range	0.002737	0.01715	0.01763	0.01722
Mean (Accuracy)	0.9926	0.9773	0.9633	0.9435
Std. Deviation	0.001098	0.0057	0.005878	0.005367
Std. Error of Mean	0.0002455	0.001275	0.001314	0.0012
Lower 95% CI of mean	0.9921	0.9746	0.9606	0.9409
Upper 95% CI of mean	0.9931	0.98	0.9661	0.946
Coefficient of variation	0.1106%	0.5832%	0.6102%	0.5688%
Geometric mean	0.9926	0.9773	0.9633	0.9434
Geometric SD factor	1.001	1.006	1.006	1.006
Lower 95% CI of geo. mean	0.9921	0.9746	0.9606	0.9409
Upper 95% CI of geo. mean	0.9931	0.98	0.9661	0.946
Harmonic mean	0.9926	0.9773	0.9633	0.9434
Lower 95% CI of harm. mean	0.9921	0.9746	0.9606	0.9409
Upper 95% CI of harm. mean	0.9931	0.9799	0.9661	0.9459
Lower 95% CI of quad. mean	0.9921	0.9746	0.9606	0.941
Upper 95% CI of quad. mean	0.9931	0.98	0.9661	0.946
Skewness	−0.1304	−0.1544	−0.04561	0.2562
Kurtosis	−1.721	−1.269	−1.248	−0.8069
Sum	19.85	19.55	19.27	18.87

Wilcoxon’s rank-sum and ANOVA tests are performed in this scenario to get the ASSOA + MLP algorithm’s p-values compared to SS + MLP, GWO + MLP, GA + MLP classification algorithms. These tests can indicate the significant difference between the ASSOA + MLP algorithm results and compared algorithms. The output p-values are shown in Table 12 for Wilcoxon’s rank-sum test, and in Table 11 for ANOVA test. Note that the p-values are less than 0.05, which indicates the superiority of the ASSOA + MLP algorithm and that the algorithm is statistically significant.

**TABLE 11:** ANOVA Test Results of the Proposed ASSOA + MLP Algorithm for Chest X-Ray Dataset

ANOVA table	SS	DF	MS	F (DFn, DFd)	P value
Treatment (between columns)	0.02618	3	0.008728	F (3, 76) = 359.7	P ¡ 0.0001
Residual (within columns)	0.001844	76	2.43E-05	–	–
Total	0.02803	79	–	–	–

**TABLE 12:** The ASSOA + MLP Algorithm’s p-Values in Comparison to Other Algorithms Using Wilcoxon’s Rank-Sum Based on Accuracy Metric

Compared Algorithms	SS + MLP	GWO + MLP	GA + MLP
p-value	1.11E-05	1.11E-05	1.11E-05

**FIGURE 10.**
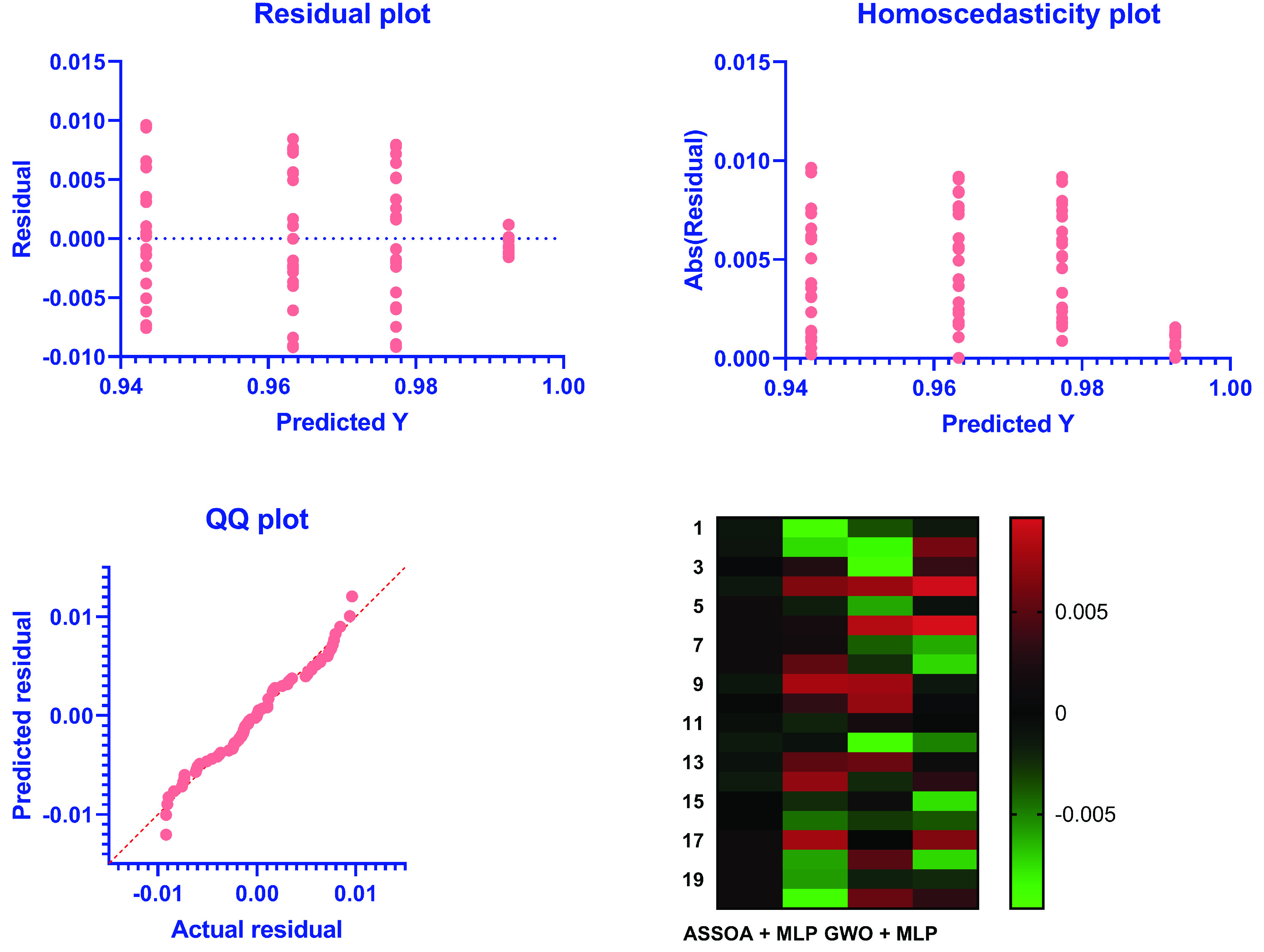
Residuals Versus Fits Plot of the proposed ASSOA + MLP algorithm compared to other algorithms for chest X-ray dataset.

The possible problems can be observed from the residual values, and residual plots rather than the original dataset plot since some datasets are not good candidates for classification. The ideal case is achieved if the residual values are equally and randomly spaced around the horizontal axis. The residual value can be calculated as (Actual value - Predicted value) with the mean and sum of the residuals are equal to zero. A residual plot is used to present the vertical axis’s residual values and the independent variable on the horizontal axis. Figure 10 shows the residual plot. A linear or a nonlinear model can be decided from plot patterns in a residual plot, and an appropriate one can be determined.

The homogeneity of variance (heteroscedasticity) provides a visual examination between the prediction errors and the predicted dependent variable scores. The heteroscedasticity plot, shown in Figure 12, can quickly determine any violation and easily improve the research findings’ accuracy. Homoscedasticity describes a situation in which the error term (random disturbance in the relationship between the dependent variable and the independent variables, or noise) is the same across the independent variables’ values.

The quantile-quantile (QQ) plot is also shown in Figure 12. It is known as a probability plot. It is mainly used by plotting their quantiles against each other to compare two probability distributions. It is noted from the figure that the points distributions in the QQ plot are approximately fit on the line. Thus, the actual and the predicted residuals are linearly related, confirming the proposed ASSOA + MLP classifier’s performance to identify the chest X-ray images.

#### 2) Chest X-Ray COVID-19 Classification Results

**FIGURE 11.**
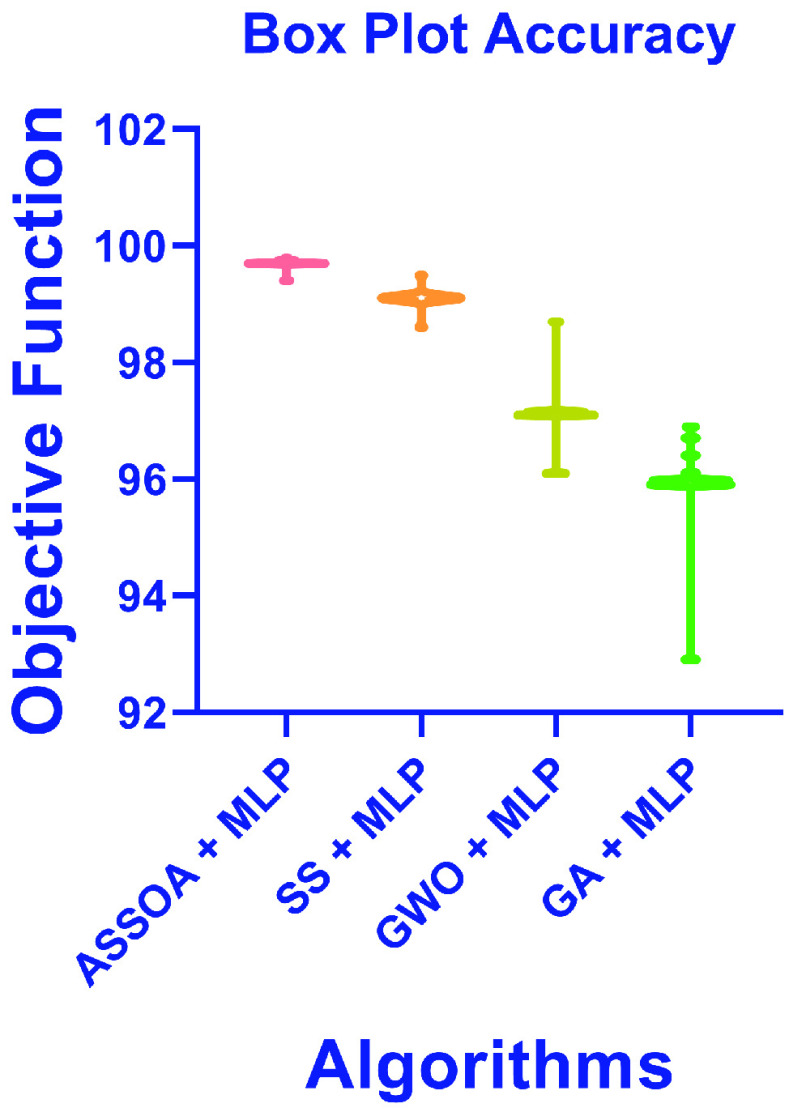
Box plot accuracy of the proposed ASSOA + MLP algorithm compared to other algorithms for chest X-ray COVID-19 dataset.

A chest X-ray COVID-19 dataset [31] is tested in the experiments to test the performance of the proposed ASSOA + MLP algorithm for the classification of chest X-ray COVID-19 cases. The output descriptive statistics of this experiment are shown in Table 13. The proposed (ASSOA + MLP) algorithm achieved a mean accuracy of (99.7%) for the COVID-19 dataset. The mean accuracy of compared algorithms of SS + MLP, GWO + MLP, and GA + MLP are (99.1%), (97.1%), and (95.9%), respectively. These results show that the proposed algorithm can improve the classification accuracy of COVID-19 patients from their chest X-ray images. The Box plot accuracy is tested, and the output figure is shown in Fig. 11. This figure shows the stability and consistency of the proposed algorithm for the classification of COVID-19 cases.

**TABLE 13:** Descriptive Statistics and the Classification Accuracy of the ASSOA Algorithm Based on MLP in Comparison to Other Algorithms for Chest X-Ray COVID-19 Images

	ASSOA + MLP	SS + MLP	GWO + MLP	GA + MLP
Number of values	20	20	20	20
Minimum (Accuracy)	0.994	0.986	0.961	0.929
25% Percentile	0.997	0.991	0.971	0.959
Median (Accuracy)	0.997	0.991	0.9712	0.9596
75% Percentile	0.9974	0.991	0.9715	0.9598
Maximum (Accuracy)	0.998	0.995	0.987	0.969
Range	0.004	0.009	0.026	0.04
Mean (Accuracy)	0.997	0.991	0.971	0.959
Std. Deviation	0.0007615	0.001478	0.00491	0.007597
Std. Error of Mean	0.0001703	0.0003305	0.001098	0.001699
Lower 95% CI of mean	0.9967	0.9903	0.9687	0.9555
Upper 95% CI of mean	0.9974	0.9917	0.9733	0.9626
Coefficient of variation	0.07638%	0.1492%	0.5057%	0.7922%
Geometric mean	0.997	0.991	0.971	0.959
Geometric SD factor	1.001	1.001	1.005	1.008
Lower 95% CI of geo. mean	0.9967	0.9903	0.9687	0.9554
Upper 95% CI of geo. mean	0.9974	0.9917	0.9733	0.9626
Harmonic mean	0.997	0.991	0.971	0.959
Lower 95% CI of harm. mean	0.9967	0.9903	0.9687	0.9553
Upper 95% CI of harm. mean	0.9974	0.9917	0.9733	0.9626
Lower 95% CI of quad. mean	0.9967	0.9903	0.9687	0.9555
Upper 95% CI of quad. mean	0.9974	0.9917	0.9733	0.9625
Skewness	−3.421	−1.099	1.029	−3.388
Kurtosis	14.35	9.79	7.08	14.38
Sum	19.94	19.82	19.42	19.18

**TABLE 14:** ANOVA Test Results of the Proposed Algorithm for Chest X-Ray COVID-19 Dataset

ANOVA table	SS	DF	MS	F (DFn, DFd)	P value
Treatment (between columns)	0.01861	3	0.006204	F (3, 76) = 293.4	P ¡ 0.0001
Residual (within columns)	0.001607	76	2.12E-05	–	–
Total	0.02022	79	–	–	–

ANOVA test is also performed for this experiment to test the ASSOA + MLP algorithm’s p-values compared to SS + MLP, GWO + MLP, GA + MLP classification algorithms. The output p-values are shown in Table 11 for the ANOVA test. Note that the p-values are less than 0.05, which indicates the superiority of the ASSOA + MLP algorithm and that the algorithm is statistically significant.

## VI. Discussion

The experiments are divided into three different scenarios to assess the proposed method performance to classify chest X-ray images. According to the promising performance, the first scenario shows that the features can be extracted from the earlier layers of the ResNet-50 model. The extracted features are fed to the next scenario for feature selection. The second scenario shows the robustness and reliability of the ASSOA algorithm in finding the optimal subset of features in a reasonable amount of time. In this scenario, Wilcoxon’s rank-sum test emphasizes the superiority of the proposed ASSOA algorithm and shows that the algorithm is statistically significant. In the third scenario, the experiments show that the proposed algorithm (ASSOA + MLP) can achieve a mean accuracy of (99.26%) and an AUC value equal to (0.9875) within (135) seconds to classify a new input X-ray chest image which outperforms other algorithms. The ASSOA + MLP algorithm also achieved a classification mean accuracy of (99.7%) for a chest X-ray COVID-19 dataset. Wilcoxon’s rank-sum and ANOVA tests confirm the proposed algorithm’s superiority and that the algorithm is statistically significant. The results and statistical tests demonstrate the high effectiveness of the proposed method in determining the infected cases.

## VII. Conclusion

Developing a classification model for diagnosing infected cases is considered one of the most critical problems, which is still much too pricey for the mass selection. This paper proposes a classification model to detect the afflicted instances from the chest X-ray images, which may dramatically minimize the diagnosis prices, particularly in cultivating nations. The training and feature extraction processes are based on a convolutional neural network (CNN) based model (ResNet50) with fine-tuning and image augmentation. The X-ray images’ classification to viral, normal, and bacterial, and popular scenarios are based upon an MLP neural network along with the proposed ASSOA algorithm. In this work, the chest X-ray images (Pneumonia) dataset composed of 5,863 X-ray images are utilized in the experiments. In the proposed model, a transfer learning technique is applied during the training stage and feature extraction. Experimental results show the proposed classification model’s efficiency in classifying the afflicted situations and a mean accuracy of (99.26%), which surpasses the cutting-edge strategies discovered in the literature. The proposed (ASSOA + MLP) algorithm also achieved a classification mean accuracy of (99.7%) for another chest X-ray COVID-19 dataset.
